# A Physiologically Relevant *In Vitro* Model of Nonreplicating Persistent *Mycobacterium tuberculosis* in Caseum

**DOI:** 10.1002/cpz1.70118

**Published:** 2025-03-08

**Authors:** Min Xie, Paulina Osiecki, Suyapa Rodriguez, Véronique Dartois, Jansy Sarathy

**Affiliations:** ^1^ Center for Discovery and Innovation Hackensack Meridian Health Nutley New Jersey; ^2^ Department of Medical Sciences Hackensack Meridian School of Medicine Nutley New Jersey

**Keywords:** caseum, drug tolerance, granuloma, *Mycobacterium tuberculosis*, nonreplicating persistence

## Abstract

Tuberculosis (TB) remains one of the leading infectious causes of death worldwide. Persistent bacterial populations in specific microenvironments within the host hamper efficient TB chemotherapy. Caseum in the necrotic core of closed granulomas and cavities of pulmonary TB patients can harbor high burdens of drug‐tolerant *Mycobacterium tuberculosis* (MTB) bacilli, making them particularly difficult to sterilize. Here, we describe protocols for the generation of a surrogate matrix using lipid‐rich macrophages to mimic the unique composition of caseum *in vivo*. Importantly, this caseum surrogate induces metabolic and physiological changes within MTB that reproduce the nonreplicating drug‐tolerant phenotype of the pathogen in the native caseous environment, making it advantageous over alternative *in vitro* models of nonreplicating persistent (NRP) MTB. The protocols include culture of THP‐1 monocytes, stimulation of lipid droplet accumulation, lysis and denaturation of the foamy macrophages, inoculation and preadaptation of MTB bacilli in the caseum surrogate, and evaluation of drug bactericidal activity against the NRP population. This novel *in vitro* model is being used to screen for potent bactericidal antimicrobial agents and to identify vulnerable drug targets, among a variety of other applications, thereby reducing our reliance on *in vivo* models. © 2025 The Author(s). Current Protocols published by Wiley Periodicals LLC.

**Basic Protocol 1**: Caseum surrogate preparation from γ‐irradiated *M. tuberculosis*–induced foamy THP‐1 monocyte–derived macrophages (THPMs)

**Alternate Protocol 1**: Caseum surrogate preparation from stearic acid–induced THPMs

**Basic Protocol 2**: Generation of nonreplicating persistent *M. tuberculosis* and drug susceptibility testing

**Alternate Protocol 2**: Higher‐throughput drug susceptibility screening using caseum surrogate

## INTRODUCTION

Tuberculosis (TB) is the leading cause of death by an infectious disease worldwide. In humans, pulmonary TB is characterized by the formation of immune cell–rich granulomas at sites of infection. Necrotic granulomas and cavities have caseous cores surrounded by cellular rims rich in macrophages, among other cell types (Basaraba & Hunter, 2017; Leong et al., 2010). These macrophages often present an abundance of lipid droplets (LDs) in the cytosol, leading to the name “foamy macrophages” (FMs) (Guerrini et al., 2018; Peyron et al., 2008). LD build‐up in FMs is attributed to a dysregulation of lipid metabolism triggered by *Mycobacterium tuberculosis* (MTB) infection and exposure to mycobacterial cell wall lipids (Kim et al., 2010; Peyron et al., 2008). Caseum itself forms as the result of accumulated necrotic debris from these FMs, creating zones of limited cellularity and avascularity within TB granulomas (Kim et al., 2010; Sarathy & Dartois, 2020). Importantly, caseum is home to extracellular nonreplicating persistent (NRP) MTB that is extremely recalcitrant to chemotherapy (Hoff et al., 2011; Lenaerts et al., 2007), making prolonged treatment durations necessary and increasing the probability of treatment failure (Sarathy & Dartois, 2020; Via et al., 2015).

Many *in vitro* NRP models, also known as “dormancy models,” have been designed to mimic environmental conditions within TB granulomas, and they have been reviewed extensively elsewhere (Dartois & Rubin, 2022; Gibson et al., 2018; Greenstein & Aldridge, 2022; Wayne & Sohaskey, 2001). Significant efforts were made to create conditions of oxygen starvation, nutrient deprivation, selective carbon sources, nitric oxide exposure, and/or low pH *in vitro*. Recent advances in the study of NRP MTB within the necrotic centers of granulomas and cavities were made possible by direct observation and manipulation of caseum from infected New Zealand White (NZW) rabbits (Sarathy et al., 2018). The *ex vivo* caseum bactericidal assay, which was developed as a reliable method to evaluate drug potency against NRP MTB in its native environment, was used to demonstrate that this subpopulation is highly phenotypically tolerant to many TB drugs (Ashwath et al., 2024; Ramey et al., 2023; Sarathy et al., 2018). The extreme drug tolerance of “fat and lazy” MTB in *ex vivo* caseum specimens despite reoxygenation and neutral pH suggests that NRP models could be redesigned to be more reflective of the caseous environment *in vivo* (Sarathy & Dartois, 2020). Furthermore, carbon starvation models do not recapitulate the abundance of triacylglycerols and cholesterol esters in these foci (Guerrini et al., 2018; Kim et al., 2010). *Ex vivo* rabbit caseum is being used routinely to evaluate the lesion‐sterilizing potential of TB drug candidates and combination regimens in the development pipeline. The integration of these potency readouts in pharmacokinetic‐pharmacodynamic (PK‐PD) translational models is enabling predictions of TB drug efficacy at physiologically achievable exposure levels at the site of disease (Ernest et al., 2021; Ramey et al., 2023; Sarathy et al., 2019; Zimmerman et al., 2017).

**Figure 1:**
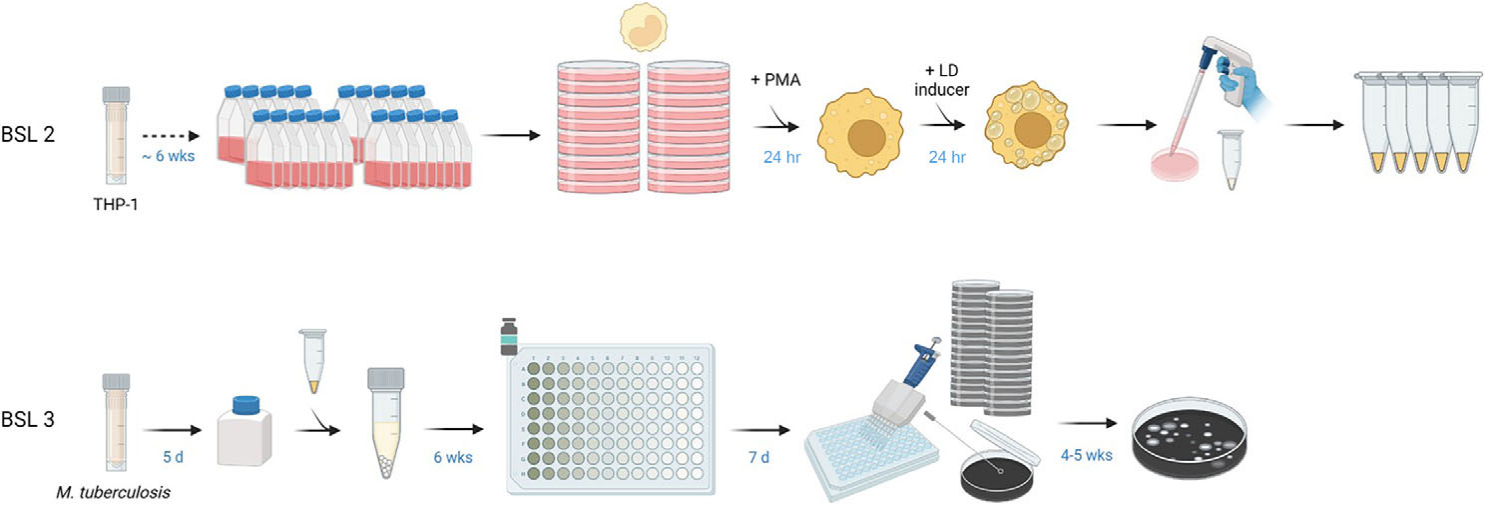
Overview of the workflow. All THP‐1 culture and manipulation are done in a Biosafety Level 2 (BSL‐2) lab under sterile conditions. Serial passaging of THP‐1 monocyte culture from a frozen vial to twenty T175 flasks provides ∼1 g caseum surrogate. Monocytes are differentiated into macrophages on large cell culture dishes. The stimulation of lipid droplet (LD) production produces foamy macrophages, which are detached, harvested, and processed for storage. All *M. tuberculosis* (MTB) culture and manipulation are done in a BSL‐3 suite. Broth cultures grown from frozen stocks, and caseum surrogate pellets are bead‐beaten together to produce a milky suspension. After a 6‐week preadaptation period, the nonreplicating bacteria suspension is used in minimum bactericidal concentration (MBC) assays. Drug potency is assessed via the enumeration of colony‐forming units (CFU) on agar plates. This figure was created with BioRender.

Unfortunately, the costs and labor‐intensiveness associated with establishing a steady supply of rabbit caseum for *ex vivo* experimentation preclude it from being used frequently and less stringently. Hence, we designed and validated an FM‐derived caseum surrogate that reproduces the biochemical composition of *ex vivo* caseum (Sarathy et al., 2023). An early version of this matrix was successfully implemented in a rapid equilibrium dialysis protocol for the measurement of caseum protein binding, a pharmacokinetic parameter that we use to infer drug penetration into these hard‐to‐treat sites of infection (Sarathy et al., 2016, 2017). However, our primary aims for the protocols being discussed here are the induction of a nonreplicating state in MTB in addition to physiological and metabolic adaptations that produce an overall increase in drug tolerance. Here, we provide detailed protocols for the *in vitro* production of caseum surrogate using two different LD inducers, providing users the flexibility to apply this model to a broad range of applications beyond the assessment of drug susceptibility in NRP MTB (see Commentary). The protocols described below can be divided into two main sections (Fig. 1). Basic Protocol 1 outlines methods for the culture and manipulation of a mammalian cell culture under Biosafety Level 2 (BSL‐2) conditions for the production of caseum surrogate, and in Alternate Protocol 1, we describe the use of stearic acid in place of irradiated MTB as an LD inducer. Basic Protocol 2 details steps for MTB preadaptation and treatment in a Biosafety Level 3 (BSL‐3) facility, and Alternate Protocol 2 describes a higher‐throughput approach to drug susceptibility testing.

*CAUTION*: MTB is a BSL‐3 pathogen and requires BSL‐3‐compliant facilities. Follow all appropriate guidelines and regulations for the use and handling of pathogenic mycobacteria. Protocols for the safe handling of MTB should be subject to consideration by the institutional biosafety committee. Work with your institution and safety officers to determine relevant personal protective equipment (PPE) and safety measures for working with MTB.

## Basic Protocol 1 CASEUM SURROGATE PREPARATION FROM γ‐IRRADIATED *M. tuberculosis*–INDUCED FOAMY THP‐1 MONOCYTE–DERIVED MACROPHAGES (THPMs)

The protocol describes a series of THP‐1 culture passages over a 6‐week period to obtain confluent growth in twenty T175 cell culture flasks, which makes ∼1 g caseum surrogate. The THP‐1 monocytic cell line is preferred for this application over peripheral blood mononuclear cells (PBMCs) due to ease of handling and greater consistency between individual batches of caseum surrogate. The culture expansion phase can be shortened or lengthened depending on the needs of the user, although it is recommended that THP‐1 cells are used at or around the same passage number to produce consistent caseum surrogate batches. Penicillin‐streptomycin may be added to THP‐1 cultures at the typical concentrations of 100 IU/ml and 100 µg/ml, respectively, to maintain sterility but should be removed from the culture medium at the final two passages and during FM generation so that these antibiotics do not interfere with MTB viability or drug tolerance in Basic Protocol 2. If anhydrotetracycline‐inducible CRISPR interference (CRISPRi) MTB libraries will be applied to the caseum surrogate model, we recommend using “Tet system–approved” fetal bovine serum (FBS), which is certified tetracycline free, during THP‐1 culture and manipulation.

**Figure 2:**
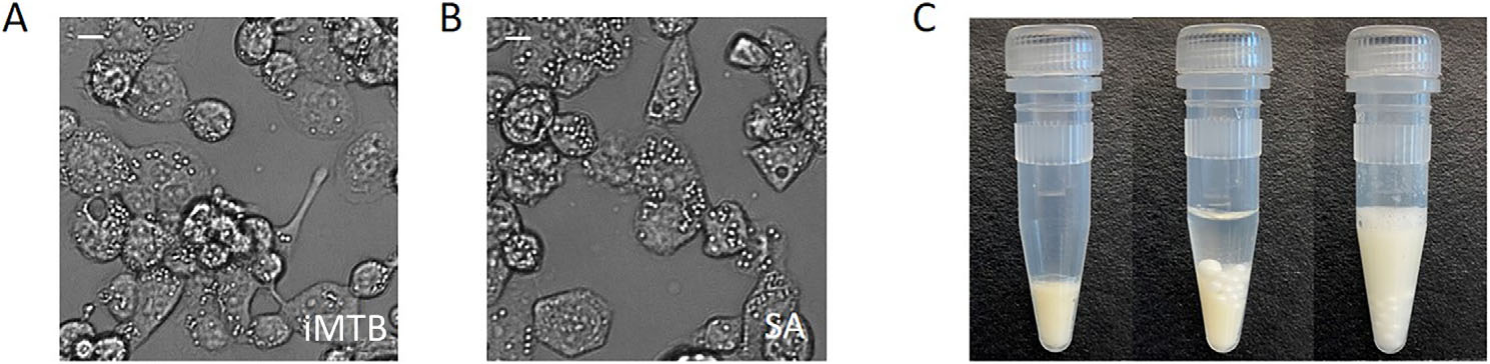
Generation and use of caseum surrogate. (**A and B**) Appearance of non‐adherent THP‐1 monocyte–derived macrophages after exposure to (A) irradiated MTB at a multiplicity of infection (MOI) of 1:50 or (B) 100 µM stearic acid. LDs appear as spherical organelles in the cytosol of foamy macrophages when visualized by bright‐field microscopy. Scale bar, 10 µm. (**C**) Appearance of caseum surrogate pellets before and after dilution with a water‐bacteria suspension. Homogenization with sterile 1.4‐mm zirconia beads produces a smooth milky suspension.

There are many validated methods for the *in vitro* generation of FMs. Fatty acids, lipoproteins, hypoxia, and exposure to MTB cell wall lipids are just some of the stimuli that induce LD accumulation in human macrophages (Bostrom et al., 2006; den Hartigh et al., 2010; Kim et al., 2010; Milosavljevic et al., 2003; Sarathy et al., 2016). Two FM inducers were validated in our work, enabling many possible applications for the surrogate matrix. γ‐irradiated MTB (iMTB) is inactivated bacteria that is safe for use in BSL‐2 labs and retains the capacity to modulate macrophage gene expression and overall immune response (Kothari et al., 2012; Madan‐Lala et al., 2011; Ting et al., 1999; Yokobori et al., 2012). The decision to use iMTB as an LD inducer stems from experimental evidence of foam cell formation in response to infection with the live bacteria and exposure to iMTB cell wall components (Russell et al., 2009). Our biochemical characterization of iMTB‐induced caseum surrogate, followed by transcriptional and microbiological evaluation of inoculated live bacteria in the said matrix, confirmed that this FM inducer creates a physiologically relevant model for the study of NRP TB (Sarathy et al., 2023). Macrophage LDs are easily visualized with a light microscope under bright field (Fig. 2A and 2B) and with the aid of fluorescent neutral lipid stains such as Nile red and Bodipy 493/503 (Greenspan et al., 1985; Sarathy et al., 2023). A total of three rinses with phosphate‐buffered saline (PBS) ensures the removal of RPMI medium components from the final matrix.

### Materials

Human monocytic leukemia cell line THP‐1 (ATCC, cat. no. TIB‐202)
Supplemented RPMI 1640 medium (see recipe), 37°C
Trypan blue solution (0.4%; Gibco, cat. no. 15250061)
100 µM PMA solution (see recipe)
iMTB suspension (see recipe)
PBS, sterile (pH 7.4; Gibco, cat. no. 10‐010‐031)
5 mM EDTA (see recipe)
Dry ice

T25 (25‐cm^2^), T75 (75‐cm^2^), and T175 (175‐cm^2^) cell culture flasks (Corning, cat. no. 431464U, 430641U, and 431080)
50‐ml centrifuge tubes (Thermo Scientific, cat. no. 339652)
Standard tabletop centrifuge (Beckman Coulter, cat. no. B99516)
1.5‐ml microcentrifuge tubes
Hemocytometer (Disposable Neubauer improved hemocytometer, SKC, cat. no. DHCN012)
Inverted microscope (Zeiss, cat. no. ZPV‐A208)
150‐mm cell culture dishes (Fisher Scientific, cat. no. FB012925)
10‐ml serological pipets (Thermo Scientific Nunc, cat. no. 170356N)
1.5‐ml screw‐cap microcentrifuge tubes, sterile (Fisher Scientific, cat. no. 02‐681‐373), pre‐weighed
37°C and 75°C heat blocks (Eppendorf, cat. no. 05‐414‐100)
Scientific balance (Mettler Toledo, cat. no. MS104S)

*NOTE*: All solutions and equipment coming into contact with cells must be sterile, and proper sterile technique should be used accordingly.

*NOTE*: All culture incubations are performed in a 37°C, 5% CO_2_ incubator (PHC, cat. no. MCO‐170AICUVL) unless otherwise specified.

1 Grow the human monocytic leukemia cell line THP‐1 in supplemented RPMI 1640 medium in a CO_2_ incubator. Starting from a frozen stock vial and a seeding density of ∼2 × 10^5^ cells/ml, sequentially expand the culture in T25, T75, and T175 cell culture flasks, with total working volumes of 10 ml, 25 ml, and 50 ml, respectively. Replace culture medium every 3 to 4 days and passage cells once a week or when the approximate cell count exceeds 8 × 10^5^ cells/ml.
  Growing twenty 175 flasks over 6 weeks will produce ∼1 g caseum surrogate. The addition of penicillin‐streptomycin to RPMI 1640 medium should be avoided to prevent any confounding effects of these antibiotics on downstream minimum bactericidal concentration (MBC) assays (see Basic Protocol 2).
2 Transfer and pool the non‐adherent THP‐1 into 50‐ml centrifuge tubes and centrifuge 5 min at 200 × *g*. Remove the spent cell culture medium and resuspend cells in 50 ml fresh supplemented RPMI 1640 medium.
3 In a 1.5‐ml microcentrifuge tube, mix 5 µl of the cell culture with 45 µl trypan blue solution to achieve a 10‐fold dilution. Transfer 10 µl to a hemocytometer and place it under an inverted microscope.
4 Count the number of viable cells within the appropriate squares of the counting grid. Calculate the total viable cell concentration, where the dilution factor (DF) is 10 if the cell density was adjusted as suggested in step 3.
  A healthy THP‐1 culture is one where the majority of the cells exclude trypan blue.
  Total cell count/ml = No. of cells per square × 10^4^ × DF
5 Add more fresh supplemented RPMI 1640 medium to adjust the cell concentration to 1.25 × 10^6^ cells/ml. Add 100 µM PMA solution to a final concentration of 100 nM and mix well by swirling.
6 Transfer 50 × 10^6^ cells, or 40 ml of the culture in step 5, to each 150‐mm cell culture dish. Transfer the dishes to the CO_2_ incubator for 24 hr.
  The non‐adherent THP‐1 monocytes will differentiate into adherent macrophages during the overnight incubation. Use a light microscope at 40× magnification for a quick visual confirmation of THPM adherence to dishes.
7 Replace the spent medium with fresh supplemented RPMI 1640 medium containing iMTB suspension at a final multiplicity of infection (MOI) of 1:50. Return the dishes to the CO_2_ incubator for 24 hr.
  iMTB, like live mycobacteria, have the tendency to clump. In order to de‐clump iMTB, stand the thawed stock vial in a water bath sonicator (Fisher Scientific, cat. no. 15337419) for 5 min and vortex well using a vortex mixer (Fisher Scientific, cat. no. 14‐955‐151) prior to use.
  If the iMTB stock suspension is prepared as suggested in the Reagents and Solution section (1.25 × 10^8^ dead cells/ml), dilute the iMTB 5000× in RPMI medium to achieve the desired burden. When working with multiple THPM dishes, the preparation of a large batch of RPMI medium plus iMTB is advised (e.g., 800 ml RPMI medium plus 160 µl iMTB stock for twenty 150‐mm dishes).
  A dish of 50 × 10^6^ macrophages should receive ∼1 × 10^6^ dead bacilli.
  The THP‐1 macrophages will accumulate LDs within the cytosol (Fig. 2A).
8 Remove the spent medium and rinse the adherent macrophages twice with 20 ml sterile PBS. Discard the rinse solutions.
9 Add 20 ml of 5 mM EDTA to each cell culture dish and incubate at 37°C for 20 min. After incubation, using a 10‐ml serological pipet, mechanically detach the macrophages by pipetting the suspension up and down repeatedly over the entire surface of the cell culture dish.
  A cell scraper (Thermo Scientific, cat. no. 179693) can also be used to gently detach the macrophages. The FMs are sensitive to extensive mechanical agitation and lyse easily.
10 Collect the cell suspension in 50‐ml tubes and centrifuge 5 min at 200 × *g*. Remove the supernatant and resuspend the cells in a total of 50 ml sterile PBS, pooling all the cell pellets into a single tube. Spin down the cells one more time and remove the supernatant.
11 Resuspend the cell pellet in 5 ml sterile PBS. Divide the suspension equally into pre‐weighed sterile 1.5‐ml screw‐cap microcentrifuge tubes. Spin down the pellets, remove all the supernatant, and store the cell pellets at –20°C.
12 Lyse the cells during three freeze‐thaw cycles by alternating the pellets between 20‐min phases in dry ice and a 37°C heat block.
13 Denature the proteins in the matrix during a 30‐min incubation at 75°C in a heat block.
  Residual PBS content can be evaporated during this period by leaving the tubes uncapped. Do not over‐dry the caseum surrogate.
14 Record the weights of the caseum surrogate pellets. Store the caseum surrogate at –20°C.
  Figure 2C illustrates the off‐white appearance of the final caseum surrogate pellets.

## Alternate Protocol 1 CASEUM SURROGATE PREPARATION FROM STEARIC ACID–INDUCED THPMs

Stearic acid is a suitable alternative for applications where the exclusion of contaminating MTB proteins, lipids, and genetic material is necessary (Sarathy et al., 2023). The saturated fatty acid (18:0) has been shown to induce LD accumulation in human monocytes (den Hartigh et al., 2010) and forms a significant proportion of the triglyceride pool of serum‐matured macrophages (Agarwal et al., 2020). Figure 2B illustrates the level of LD accumulation in THPMs following 24‐hr exposure to 100 µM stearic acid.

### Additional Materials (also see Basic Protocol 1)

Supplemented RPMI 1640 medium (see recipe) containing 100 µM stearic acid (diluted from 100 mM stearic acid solution; see recipe), 37°C

*NOTE*: Stearic acid is poorly soluble in aqueous medium. Pre‐warm the RPMI 1640 medium before use to improve solubility. Mix the RPMI medium well by swirling after the addition of 100 µM stearic acid to disperse the whitish precipitate. Any remaining precipitate will dissolve during the overnight incubation at 37°C (see step 1) and will not compromise the experiment.

1 Follow steps 1 to 6 in Basic Protocol 1 to generate cell culture dishes with THP‐1 monocyte–derived macrophages. After 24 hr of exposure to PMA, replace the cell culture medium with fresh supplemented RPMI 1640 medium containing 100 µM stearic acid. Return the dishes to the CO_2_ incubator for 24 hr.
  If the stearic acid stock solution is prepared as suggested in the Reagents and Solutions section (100 mM), dilute it 1000× in RPMI medium to achieve the desired concentration. When working with multiple THPM dishes, the preparation of a large batch of RPMI medium plus stearic acid is advised (e.g., 800 ml RPMI medium plus 800 µl stearic acid stock solution for twenty 150‐mm dishes).
  The THP‐1 macrophages will accumulate LDs within their cytosol (Fig. 2B).
2 Follow steps 8 to 14 in Basic Protocol 1 to harvest, lyse, denature, and store the FM‐derived caseum surrogate.

## Basic Protocol 2 GENERATION OF NONREPLICATING PERSISTENT *M. tuberculosis* AND DRUG SUSCEPTIBILITY TESTING

**Figure 3:**
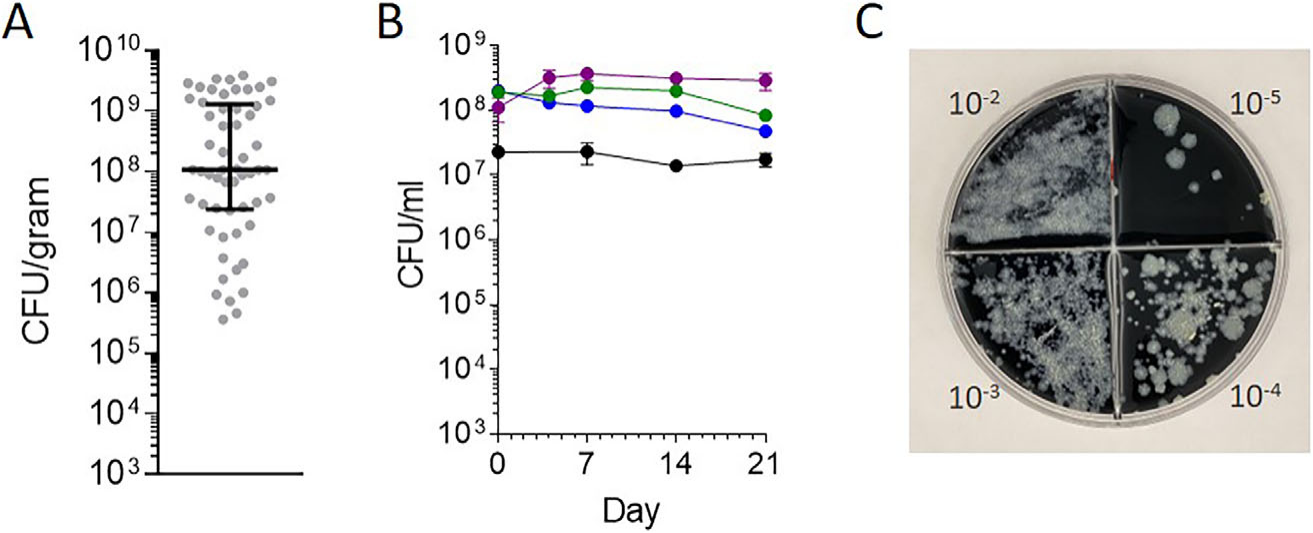
Bacterial burdens and strains. (**A**) MTB burdens in 57 independent *ex vivo* rabbit caseum specimens (gray dots). Black lines indicate the median and interquartile range. (**B**) The growth kinetics of different wild‐type MTB lab strains in caseum surrogate indicate that these bacterial populations maintain a viable but nonreplicating state. Inoculated wild‐type HN878 (green), Erdman (blue), and H37Rv (purple) MTB strains in surrogate were compared to the native HN878 population of *ex vivo* rabbit caseum (black). (**C**) Ten‐fold serial dilutions of caseum surrogate from each well are spread onto quadrant charcoal agar plates such that each whole plate permits CFU enumeration within a 4‐log window, limiting the amount of resources needed to quantify a wide range of bacterial burdens.

MTB from replicating broth cultures quickly adopts the nonreplicating state upon inoculation into caseum surrogate (Basic Protocol 1 and Alternate Protocol 1). The inoculation of MTB culture at the adjusted optical density (600 nm) of 0.8 provides a caseum surrogate suspension with an approximate bacterial burden of 10^7^ CFU/ml, which is the average burden observed in caseum specimens from infected NZW rabbits (Fig. 3A) (Sarathy et al., 2018). Furthermore, we discuss the inoculation of wild‐type MTB HN878 because this strain is used in the rabbit model of chronic pulmonary cavitary TB (Blanc et al., 2018; Subbian et al., 2011). It is therefore present in caseum specimens used in the *ex vivo* MBC assay that was used to calibrate the caseum surrogate model during method validation (Sarathy et al., 2018, 2023). However, the protocol is suitable for use with other wild‐type MTB strains as well (Fig. 3B).

**Figure 4:**
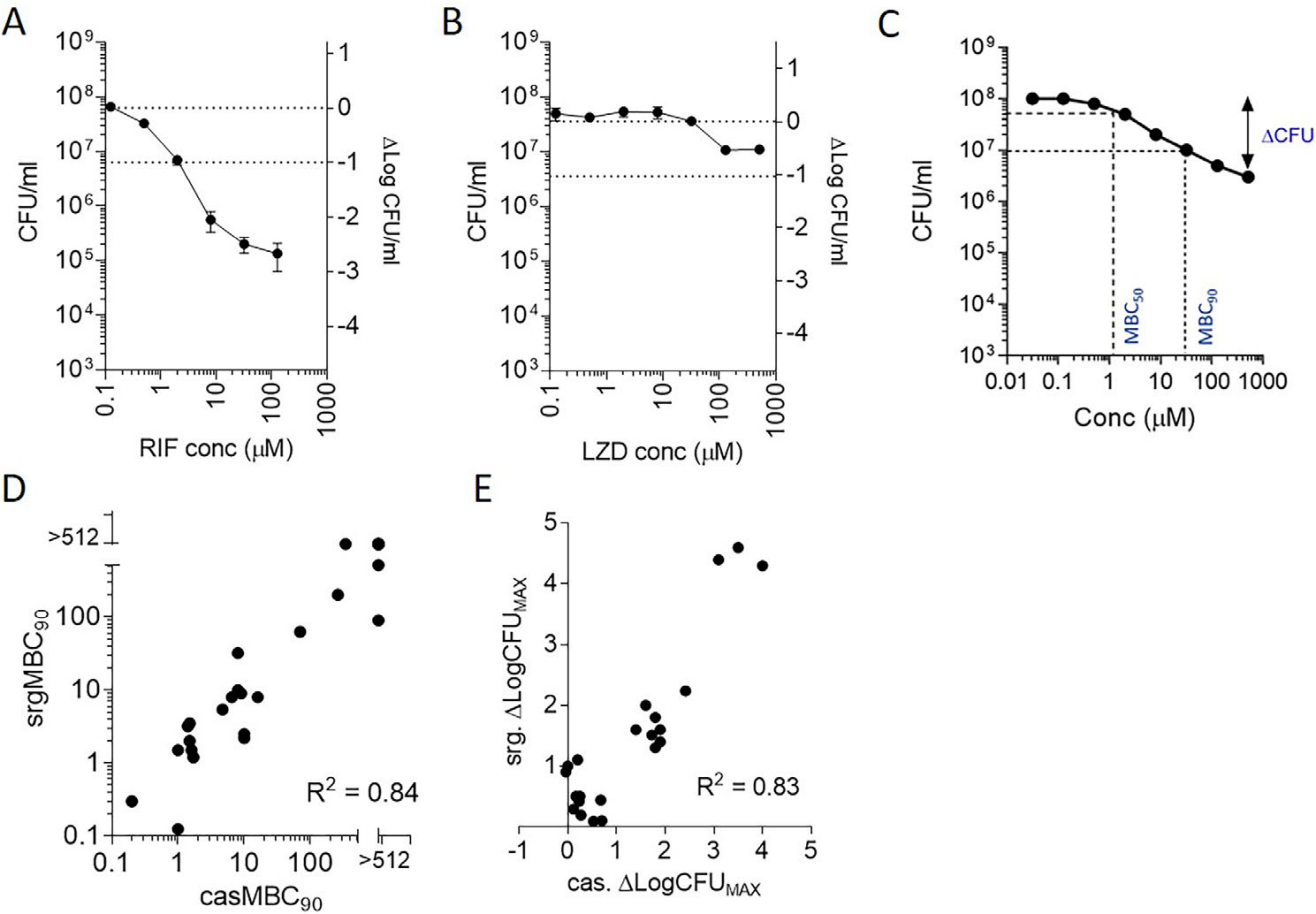
Potency readouts in caseum surrogate. (**A and B**) Representative bactericidal activity dose‐response curves for potent (A; rifampicin, RIF) and weakly active (B; linezolid, LZD) tuberculosis drugs. Absolute bacterial burdens (left *y*‐axis), net changes in burden (right *y*‐axis), and drug concentrations (*x*‐axis) are plotted on logarithmic scales. Dotted lines indicate the burdens in control wells and cutoffs for 90% CFU reduction (surMBC_90_). (**C**) A mock dose‐response curve illustrates how MBC_50_, MBC_90_, and ΔCFU readouts are obtained. (**D**) Correlation between bactericidal activity in caseum (casMBC_90_) and in irradiated MTB*‐*induced surrogate (iMTB‐srgMBC_90_). (**E**) Correlation between maximal CFU reduction at the highest assay concentration (Δlog CFU_512µM_) in caseum (cas) and surrogate (srg). Panels (D) and (E) were reproduced from Sarathy et al. (2023) with permission.

The second section of this protocol describes a bactericidal assay conducted in a 96‐well format with colony enumeration as the final readout. However, it can be scaled differently and optimized for alternative readout formats. Although our method provides full dose‐response curves (Fig. 4A and 4B), the caseum surrogate bactericidal assay can also be utilized for higher‐throughput single‐point screens of compounds in earlier stages of drug development (Alternate Protocol 2). The dilution of drug solutions and assay plate spotting are done manually using the sample dilution scheme in Table 1. Digital dispensers such as the Tecan D300e support rapid yet accurate establishment of dose‐response curves, with the caveat that they typically do not handle dimethyl sulfoxide (DMSO) stock solutions with concentrations >10 mM. Serial four‐fold dilutions instead of the typical two‐fold ones permit the study of drug potency over a wide concentration range within an eight‐point dose‐response assay. The high drug concentrations assayed in this protocol can lead to significant carryover levels on 7H11 agar plates, dampening of colony growth, and overestimation of drug potency. To circumvent this problem, activated charcoal (0.4%) is added to the agar to absorb drug carryover. Charcoal powder (carbon) does slow mycobacteria colony growth and reduces the selective nature of Middlebrook 7H11 medium by facilitating fungal growth (Suzuki & Iwahashi, 2016). Longer colony growth periods (4 weeks) and the addition of trimethoprim, cycloheximide, and polymyxin B to charcoal–7H11 agar produce optimal MTB colony growth. This protocol discusses the derivation of MBC_50_ and MBC_90_ readouts, which refer to the minimum drug concentrations that achieve 50% or 90% NRP TB killing, respectively (Fig. 4C). It also considers the total reduction in bacterial burden at the maximum drug concentration tested, from which we infer the sterilizing potential of the drug or drug candidate (Sarathy et al., 2023).

**Table 1:** Drug Dilution Scheme and Bactericidal Assay Plate Preparation

Drug dilution plate			MBC assay plate		
Well	Serial dilutions	Working stock conc.	Vol. of drug solution	Surrogate‐MTB suspension	Final assay conc.
A	20.5 µl 50 mM stock + 19.5 µl DMSO	25,600 µM	1 µl of A	50 µl	512 µM
B	10 µl of A + 30 µl DMSO	6400 µM	1 µl of B	50 µl	128 µM
C	10 µl of B + 30 µl DMSO	1600 µM	1 µl of C	50 µl	32 µM
D	10 µl of C + 30 µl DMSO	400 µM	1 µl of D	50 µl	8 µM
E	10 µl of D + 30 µl DMSO	100 µM	1 µl of E	50 µl	2 µM
F	10 µl of E + 30 µl DMSO	25 µM	1 µl of F	50 µl	0.5 µM
G	10 µl of F + 30 µl DMSO	6.25 µM	1 µl of G	50 µl	0.125 µM
H	10 µl of G + 30 µl DMSO	1.56 µM	1 µl of H	50 µl	0.03125 µM
‐	n/a	n/a	1 µl of DMSO	50 µl	0

### Materials

Supplemented 7H9 broth medium (see recipe), 37°C
MTB HN878 (BEI Resources, cat. no. NR‐13647)
Molecular–grade water, sterile (Invitrogen, cat. no. 10977‐015)
Caseum surrogate pellets (see Basic Protocol 1)
Test TB drugs
DMSO (Sigma, cat. no. 472301) or other appropriate diluent
PBS‐0.0625% Tween 80 (PBS‐Tween; see recipe)
Supplemented 7H11 agar medium plates (see recipe), 37°C

30‐ml square bottles, sterile (Nalgene square PETG media bottle, Fisher Scientific, cat. no. 22‐030595)
37°C shaker incubator (Chemglass Life Sciences, cat. no. CLS2543001)
Cuvette (Fisher Scientific, cat. no. 14‐955‐127)
Spectrometer (Avantor, cat. no. 490005‐906)
2‐ml screw‐cap microcentrifuge tubes
Microcentrifuge (Eppendorf, cat. no. 5424R)
1.4‐mm zirconia beads, sterile (Cayman Chemical, cat. no. 10402)
Bead Mill Homogenizer (Bead Mill 24 Homogenizer, Fisherbrand, cat. no. 15‐340‐163)
1.5‐ml screw‐cap microcentrifuge tubes with O‐rings (Fisher Scientific, cat. no. 02‐681‐373)
37°C incubator
Clear 96‐well round‐bottom plates (Corning, cat. no. 3788)
Clear 96‐well flat‐bottom plate (Corning, cat. no. 3370)
Vortex mixer (Fisher Scientific, cat. no. 02‐215‐418)
Non‐breathable adhesive sealing film (Thermo Scientific, cat. no. 232701)
Inoculating loops

### MTB inoculation and preadaptation

1 Inoculate 10 ml supplemented 7H9 broth medium with MTB HN878 in a sterile 30‐ml square bottle. Incubate at 37°C for 5 days with shaking at 80 rpm.
  MTB HN878, H37Rv, or Erdman strains can be used, depending on the purpose of the study. The inoculum volume depends on the density of the frozen stock. Selection antibiotics can be added to the broth culture when growing genetically modified strains but should be washed out prior to the inoculation of caseum surrogate.
2 Measure the optical density of the culture at 600 nm (OD_600_) in a cuvette with a spectrometer.
  The bacterial culture should be in the exponential growth phase, within the ideal OD_600_ range of 0.6 to 0.9.
3 Transfer 1 ml of the bacterial culture to a 2‐ml screw‐cap microcentrifuge tube and spin down 5 min at 13,000 × *g* in a microcentrifuge. Remove the supernatant and resuspend the pellet in the appropriate amount of molecular–grade water such that an approximate OD_600_ of 0.8 is achieved.
  The density of the MTB culture can be adjusted depending on the purpose of the study. Adjusting the OD_600_ to 0.8 prior to inoculation of the caseum surrogate ensures a starting bacterial density that is reflective of ex vivo rabbit caseum (i.e., 10^7^ to 10^8^; Fig. 3A) (Sarathy et al., 2018).
4 Considering the weight of the caseum surrogate pellet in each tube, add two volumes of the bacteria‐water suspension such that the final dilution factor is three‐fold (w/v).
5 Add approximately 10 sterile 1.4‐mm zirconia beads to each tube and homogenize in a single cycle in a bead mill homogenizer at speed setting 5.0 for 30 s.
  Alternative homogenizers to the Beal Mill 24 Homogenizer with a similar speed setting are permissible as long as the diluted caseum surrogate is a milky homogenous suspension (Fig. 2C). Simply vortexing the tube will not produce a fine suspension.
6 Sample 20 µl of the suspension, perform 10‐fold serial dilutions with PBS‐Tween, and spread on agar plates for colony‐forming unit (CFU) enumeration as described in steps 14 to 16.
  This allows the user to establish the exact bacterial burden on the day of inoculation prior to the preadaptation phase.
7 Pool caseum surrogate homogenates into new 1.5‐ml screw‐cap microcentrifuge tubes with O‐rings. Incubate in 37°C incubator for 6 weeks without agitation.
  Avoid pooling >1 ml homogenate per 1.5‐ml tube to maintain sufficient air headspace. The homogenate settles over 6 weeks, but constant mixing is not necessary.

### Bactericidal activity assay

8 Prepare 50 mM test TB drug stock solutions in DMSO or another appropriate diluent and store these at –20°C.
  Filter stock solutions made with water through a 0.22‐µm filter unit to ensure sterility.
9 Before starting the bactericidal assay, thaw and make serial dilutions of 50 mM drug stock solutions in their corresponding vehicles in tandem in a clear 96‐well round‐bottom plate.
  The series of working drug dilutions should be 50‐fold higher than the desired final test concentrations in the bactericidal assay. A sample dilution scheme is provided in Table 1.
10 Pipet 1 µl of each working drug dilution into a clear 96‐well flat‐bottom plate. Include one well containing 1 µl vehicle only.
11 Vortex the bacteria‐surrogate suspension after the 6 weeks of preadaptation (see step 7). Then, sample 20 µl of the suspension, perform 10‐fold serial dilutions with PBS‐Tween, and spread on agar plates for CFU enumeration as described in steps 14 to 16.
  This allows the user to establish the exact bacterial burden of the suspension post‐preadaptation and prior to the MBC assay.
12 Aliquot 50 µl bacteria‐surrogate suspension per well containing diluted drug solution or vehicle only (see step 10).
  The suggested final drug concentration range is 0.03125 to 512 µM.
13 Seal the plate with a non‐breathable adhesive sealing film. Incubate the plate at 37°C for 7 days without shaking.
14 Open the sealing film and use a pipet to resuspend the caseum surrogate suspension in each well. Transfer 20 µl from each well into a clear 96‐well round‐bottom plate pre‐filled with 180 µl PBS‐Tween per well. Perform 10‐fold serial dilutions of the caseum surrogate samples.
15 Spread the appropriate dilutions on supplemented 7H11 agar medium plates, with 25 µl diluted sample per quadrant spread with an inoculating loop. Incubate the agar plates at 37°C for 4 weeks.
  Figure 3C illustrates an efficient CFU plating scheme that allows for each agar plate to have maximal coverage over a 4‐log dilution range, so that the user obtains enumerable colony growth with the fewest agar plates. Each condition is plated on triplicate agar plates.
  To ensure the recovery of enumerable CFU, the dilution range plated on agar should be adjusted depending on the expected potency of the drug at that specific concentration. Vehicle‐control wells and low‐drug‐concentration wells should be plated up to the 10^5^ dilution, whereas dilutions ≤10^3^ are more appropriate for drug wells where significant cidal activity is predicted.
16 Count and record the number of CFU in each quadrant containing fewer than 100 colonies.

### Calculations

17 Determine the viable number of MTB in each well of the MBC assay by multiplying the CFU with the respective dilution factor (10^n^) for that quadrant.
  Given that 25 µl was spread on each agar quadrant in step 15, the bacterial burden per ml of homogenate can be calculated by further multiplying the counts by a factor of 40:
  CFU × 10^n^ × 40 = Viable MTB / ml
18 Plot the viable MTB counts against drug concentration, as shown in Figures 4A and 4B. Using the viable MTB counts from the vehicle‐only control well, calculate the cutoff for 90% bacterial killing or a 1‐log reduction in burden. On each dose‐response curve, determine the minimum drug concentration that achieves 90% bacterial killing (surMBC_90_), as illustrated in Figure 4C.
19 To calculate the Δlog CFU_max_, first calculate the logarithm of the CFU/ml counts in the DMSO‐only control well and 512 µM drug well, with a base of 10. Then, subtract the former from the latter.
  ΔLog CFU_max_ = Log(CFU/ml_512µM_) – Log(CFU/ml_control_)

## Alternate Protocol 2 HIGHER‐THROUGHPUT DRUG SUSCEPTIBILITY SCREENING USING CASEUM SURROGATE

**Figure 5:**
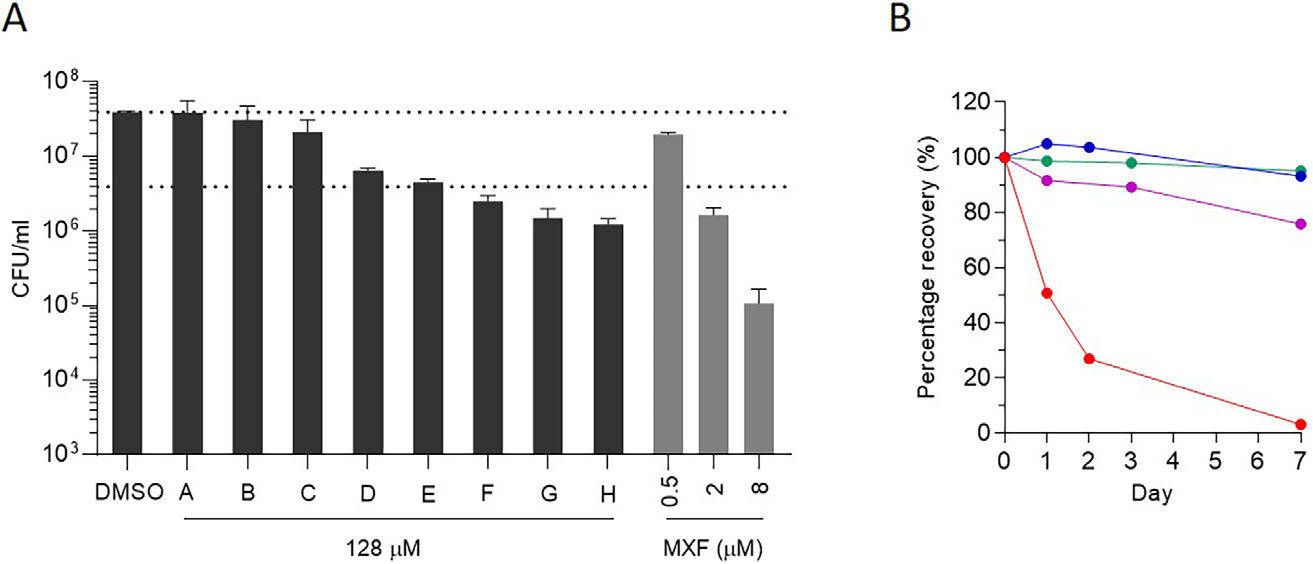
Single‐point screens and chemical stability. (**A**) Mock‐up of a compound screen of eight hypothetical drug candidates with varying potency in the surrogate caseum MBC assay in the single‐point format. Gray bars illustrate how moxifloxacin (MXF) actually performs as an internal control at the incubation concentrations of 0.5, 2, and 8 µM. Dotted lines indicate the burdens in control wells and cutoffs for 90% CFU reduction (surMBC_90_). (**B**) Percentage recovery (%) of four representative drugs in caseum surrogate over 7 days at 37°C. Para‐aminosalicylic acid (blue), a de‐identified menaquinone biosynthesis inhibitor (green), a de‐identified ATP synthase inhibitor (purple), and delamanid (red) were incubated in three‐fold‐diluted caseum surrogate at the starting concentration of 5 µM. Samples were processed for quantitative analysis by liquid chromatography coupled to tandem mass spectrometry.

Although drug potency testing in caseum surrogate typically takes the form of full dose‐response curves, as illustrated in Figures 4A and 4B, the protocol can be amended to permit higher‐throughput testing of compounds in single‐ or dual‐point formats. In an initial assessment of relative potency, drug candidates are tested in parallel at the single high concentration of 128 µM (Fig. 5A). This assay format allows for the quick identification and exclusion of compounds that are simply inactive against NRP TB, thereby saving resources and time. Figure 5A also illustrates how moxifloxacin, a second‐line TB agent, is used as a positive control in this model during drug screening, ensuring consistency between experiments.

### Additional Materials (also see Basic Protocol 2)

Appropriate internal control (e.g., moxifloxacin)

1 Follow steps 1 to 7 in Basic Protocol 2 to generate NRP MTB in the caseum surrogate model. Using 50 mM stock solutions of the test inhibitors and the dilution scheme in Table 1, prepare a 128 µM test well for each of the compounds to be tested in a single‐point screening assay. If a dual‐point screen is desired (i.e., with 128 and 512 µM), also use Table 1 to prepare the appropriate working solutions representing two inhibitor concentrations for each compound. Pipet 1 µl of each drug stock dilution into a 96‐well flat‐bottom plate. Include one well containing 1 µl vehicle only. Also include an appropriate internal control, such as moxifloxacin, as shown in Figure 5A.
2 Follow steps 11 to 18 in Basic Protocol 2 to determine the bactericidal activity of each test inhibitor.

## REAGENTS AND SOLUTIONS

### γ‐irradiated M. tuberculosis (iMTB) suspension

γ‐irradiated *M. tuberculosis* HN878 (BEI Resources, cat. no. NR‐49100)
Resuspend iMTB pellet in PBS (Gibco, cat. no. 10‐010‐031)
De‐clump suspension by repeated pipetting and using water bath sonicator (Fisher Scientific, cat. no. 15337419) for 10 min
Enumerate density under light microscope at 1000× magnification
Adjust density with PBS to ∼1.25 × 10^8^ iMTB bacilli/ml such that it is 5000× diluted in supplemented RPMI 1640 medium prior to its introduction to THPMs and a final MOI of 1:50 is achieved
Store ≤2 years at –80°C

### EDTA, 5 mM

10 ml 0.5 M ethylenediaminetetraacetic acid (EDTA; Invitrogen, cat. no. AM9262)
1 L PBS (Gibco, cat. no. 10‐010‐031)
Store ≤1 month at room temperature

### Glycerol, 50%

100 g glycerol (MilliporeSigma, cat. no. GX0185)
100 ml deionized water
Stir with magnetic stir bar on stirrer plate for 30 min, until completely mixed
Filter‐sterilize with 0.22‐µm filter unit (250‐ml filter unit, Thermo Scientific, cat. no. 5680020)
Store ≤1 year at room temperature

### PBS‐0.0625% Tween 80 (PBS‐Tween)

625 µl 20% (w/v) Tween 80 (see recipe)
1 L PBS (Gibco, cat. no. 10‐010‐031)
Store ≤6 months at 4°C, protected from light

### PMA solution, 100 µM

1 mg phorbol 12‐myristate 13‐acetate (PMA; Fisher BioReagents, cat. no. BP685‐1)
16.2 ml 200‐proof ethanol (Fisher Chemical, cat. no. BP2818‐4)
Mix well
Store ≤6 months at 4°C, protected from light

### Stearic acid solution, 100 mM

284 mg stearic acid powder (Alfa Aesar, cat. no. A12244)
10 ml 200‐proof ethanol (Fisher Chemical, cat. no. BP2818‐4)
Mix well
Store ≤6 months at 4°C
  Stearic acid recrystallizes at low temperatures but dissolves again when warmed to 37°C prior to use.

### Supplemented 7H11 agar medium

10 g Middlebrook 7H11 powder (Remel, cat. no. R454002)
450 ml deionized water
5 ml 50% (w/v) glycerol (see recipe)
2 g activated charcoal (Sigma‐Aldrich, cat. no. C9157)
Autoclave at 121°C for 30 min
Cool to ∼55°C
Add 50 ml oleic acid‐albumin‐dextrose‐catalase (OADC; Remel, cat. no. R450605)
Mix well
Pour agar into 100‐mm quadrant plates (100 × 15 mm; Fisherbrand, cat. no. FB087582) immediately (25 ml per whole dish or 6 ml per quadrant)
Allow agar to solidify at room temperature overnight
Store ≤4 weeks at 4°C
  The final concentrations in the medium are 0.5% (w/v) glycerol, 0.4% (w/v) activated charcoal, and 10% (v/v) OADC.

### Supplemented 7H9 broth medium

4.7 g Middlebrook 7H9 powder (Sigma‐Aldrich, cat. no. M0178‐500G)
900 ml deionized water
4 ml 50% (w/v) glycerol (see recipe)
2.5 ml 20% (w/v) Tween 80 (see recipe)
100 ml albumin‐dextrose‐catalase (ADC; Becton Dickinson, cat. no. 212352)
Mix well
Filter‐sterilize with 0.22‐µm filter unit (1‐L filter unit, Thermo Scientific, cat. no. 5670020)
Store ≤4 weeks at 4°C
  The final concentrations in the medium are 0.2% (w/v) glycerol, 0.05% (w/v) Tween 80, and 10% (v/v) ADC.

### Supplemented RPMI 1640 medium

1 L RPMI 1640 medium plus 2 mM l‐glutamine and phenol red (Gibco, cat. no. 11875135)
100 ml heat‐inactivated FBS (Gibco, cat. no. 16140‐071)
Store ≤4 weeks at 4°C

### Tween 80, 20%

20 g Tween 80 (Fisher Chemical, cat. no. T164‐500)
80 ml deionized water
Stir with magnetic stir bar on stirrer plate for 2 hr, until completely mixed
Filter‐sterilize with 0.22‐µm filter unit (250‐ml filter unit, Thermo Scientific, cat. no. 5680020)
Store ≤6 months at 4°C, protected from light
  Protect from light during both preparation and storage.

## COMMENTARY

### Background Information

The approach described here was originally validated for use as a drug screening tool to identify TB drugs with granuloma‐sterilizing potential (Sarathy et al., 2023). Overall, caseum surrogate is supporting screening for new bactericidal compounds, the identification of vulnerable drug targets, the evaluation of synergistic drug‐drug interactions in multidrug regimens, and the unravelling of transcriptomic changes in dormant bacteria. By monitoring the viability and phenotype of knockout mutants and complement strains in lipid‐rich caseum surrogate, the model is used to evaluate gene function in NRP bacteria, as was recently demonstrated with the MTB protein CinA (Kreutzfeldt et al., 2022). Caseum surrogate was recently used in the quantification of the rRNA synthesis (RS) ratio, a pharmacodynamic marker that represents ongoing RS, to study antibiotic response in MTB subpopulations from different lung microenvironments (Walter et al., 2023). Ongoing experiments with anhydrotetracycline‐inducible CRISPRi libraries in caseum surrogate are attempting to identify novel drug targets that are critical to MTB survival in host niches (Li et al., 2022; Rock, 2019). Furthermore, this NRP model is applicable to other nontuberculous mycobacteria (NTM) strains. The approach was used to demonstrate the tolerance of nonreplicating *Mycobacterium abscessus*, an opportunistic pathogen, to multiple drug classes (Xie et al., 2023). To the best of our knowledge, the present article describes the first protocol (Basic Protocol 1) for generating a cell‐based matrix that mimics caseum for use in an *in vitro* NRP MTB model.

### Critical Parameters

#### Scale

The total mass of THPMs harvested using Basic Protocol 1 will vary depending on the total number of THP‐1 monocytes applied at the start. In our hands, twenty 175 flasks with dense THP‐1 cultures hold a total of ∼1 × 10^9^ monocytes, which form dense THPM monolayers on twenty 150‐mm dishes. This input level consistently produces approximately 0.8 to 1 g caseum surrogate. The protocol should be scaled to meet the needs of the user. This protocol is amenable to alternative cell culture flasks and dishes and culture passage schedules. With regards to Basic Protocol 2, 500 mg caseum surrogate allows for the preparation of 30 test and control wells in bactericidal activity assay(s) when using the exact conditions mentioned above. Caseum surrogate pellets should be prepared and stored such that they are easily plugged into the specific routine application. For instance, running two eight‐point full dose‐response curves at a go in the bactericidal activity assay described above requires ∼300 mg caseum surrogate. Hence, this specific workflow benefits from the storage of caseum surrogate pellets in the weight range of 100 to 200 mg, to be pooled as needed.

#### Cell passage number

In the interest of obtaining reproducible drug susceptibility measurements from consistent batches of caseum surrogate, we advise using THP‐1 monocytes at the same passage number for each experiment. In Basic Protocol 1, we described the subculture of THP‐1 cells from a frozen stock vial six times, obtaining 20 dense T175 flasks within 7 weeks, before use in step 2.

#### Batch preparation of LD inducer

In order to further promote consistency, the iMTB stock suspension (Basic Protocol 1) and stearic acid stock solution (Alternate Protocol 1) should be prepared in large batches; aliquots can be stored at –20°C and 4°C, respectively.

#### Undisturbed preadaptation phase

The “preadaptation phase” (Basic Protocol 2) refers to the 6‐week period during which MTB develops an NRP phenotype in caseum surrogate. During this period, the system is best left undisturbed. Refrain from opening the tube or mixing the contents. Tubes with an O‐ring component limit loss of water content through evaporation during the long incubation period, as well as limiting the influx of oxygen into the system, thereby recapitulating the reduced oxygen tension of nonvascularized caseous cores of necrotic granulomas (Sarathy & Dartois, 2020). Users will observe that the bacteria‐surrogate suspension settles during this phase, but no mechanical agitation (i.e., constant shaking) is required to prevent this sedimentation.

#### Stability of chemical inhibitor

Labile compounds such as esters that are unstable in plasma are predictably unstable in caseum and the surrogate matrix as well. This instability compromises a drug's bactericidal activity, as measured in the 7‐day MBC assay with a single starting dose (Basic Protocol 2), but does not negate the drug's use in a treatment regimen where daily dosing ensures regular replenishment of tissue drug levels. This caveat is exemplified by the drug delamanid, which is part of a highly successful multidrug‐resistant TB treatment regimen (Conradie et al., 2020). Delamanid is rapidly degraded in plasma and tissue due to albumin‐mediated metabolism (Sasahara et al., 2015). The chemical stability of a test compound can be easily determined in a 7‐day stability assay in caseum surrogate, where liquid chromatography–mass spectrometry (LC‐MS) enables the quantitation of compound recovery over time. Figure 5B illustrates the relative instability of delamanid in caseum surrogate in comparison to three other antibiotics that exhibit negligible or acceptable levels of compound degradation over 7 days at 37°C.

### Troubleshooting

Table 2 describes possible problems that may arise from the protocols outlined here and proposed solutions.

**Table 2:** Troubleshooting Guide for the Preparation and Use of Caseum Surrogate as an *In Vitro* Model of NRP MTB

Problem	Possible reason	Solution
The compound does not completely solubilize at 50 mM	The compound is not highly soluble in DMSO	Consider alternative vehicles
		Sonicate the stock solution briefly
		Make a 10 mM stock and test drug potency up to a maximum concentration of 128 µM
Stearic acid precipitates when added to cell culture medium	The fatty acid is poorly soluble in aqueous medium	Pre‐warm the RPMI 1640 medium to improve solubility; the precipitate should dissipate gradually during the overnight incubation
The iMTB suspension is clumpy	The dead bacteria have formed aggregates	Place in a water bath sonicator for 5 min and vortex well before proceeding
The THPM cell pellet masses are much lower than expected, and the wash supernatants remain cloudy even after centrifugation	The macrophages have lysed and released their cytosolic contents	Avoid vigorous scraping; instead, use repeated gentle pipetting to resuspend FMs after EDTA treatment
The caseum surrogate–bacteria suspension partially evaporates during the long preadaptation phase	The tube caps do not have a tight seal, permitting evaporation within the warm incubator	Use tubes with O‐ring rubber seals Parafilm tubes over the 6‐week incubation
The tubes containing caseum surrogate pellets have significant liquid content	A small volume of residual PBS could not be removed by pipetting without disturbing the pellets	Uncap the storage tubes during the denaturation step (75°C) to allow residual liquid to evaporate
Lack of bactericidal activity	The compound could be unstable in caseum surrogate	Perform a stability assay over 7 days in the same matrix
	The test compound is inactive against NRP MTB	Consider alternative drugs or drug targets
Extensive fungal contamination of agar plates	The 0.4% charcoal reduces the selectivity of Middlebrook 7H11 medium for mycobacteria	Add 20 µg/ml trimethoprim, 50 µg/ml cycloheximide, and 200 U/ml polymyxin B to the 7H11 agar
		Use sterile disposable lab consumables and aseptic techniques during all steps of the protocol

### Understanding Results

MTB from replicating broth cultures quickly adopts the nonreplicating state upon inoculation into caseum surrogate. Assessment of genome replication using patterns of metagenomic sequencing read coverage revealed that this transition to growth stasis in caseum surrogate takes 4 to 7 days (Sarathy et al., 2023). However, presentation of the drug‐tolerant phenotype that is representative of MTB in its native caseous environment requires a 6‐week preadaptation phase (Sarathy et al., 2023). Figure 4A illustrates the potency of rifampicin, a first‐line TB agent, in caseum surrogate, emphasizing its role as an effective sterilizing agent. Figure 4B illustrates the lack of potency of linezolid in caseum surrogate, with a typical dose‐response profile for drugs that are poorly bactericidal in this NRP model. Ultimately, our results demonstrate that caseum surrogate reproduces the drug tolerance profile of caseum MTB, as illustrated in Figure 4D, with a strong correlation in MBC_90_ measurements between the two matrices. The strong correlation between ΔlogCFU_max_ in caseum and the surrogate further emphasizes the *in vitro* model's ability to predict TB lesion sterilization potential (Fig. 4E).

### Time Considerations

See Table 3 for the approximate time needed for Basic Protocols 1 and 2. The respective time frames for Alternate Protocols 1 and 2 are the same.

**Table 3:** Time Considerations for Basic Protocols 1 and 2

Basic Protocol	Step(s)	Activity	Estimated time needed
1	1	THP‐1 cell culture	Variable; up to 6 weeks if twenty T175 flasks are expected from a single frozen stock vial
	2‐6	Preparation of THPM dishes	24 hr
	7	Addition of FM inducer	24 hr
	8‐11	Harvest of FMs	2‐3 hr
	12‐14	Lysis, denaturation, and weighing	2.5 hr
2	1	Growth of replicating MTB broth culture	5 days
	2‐6	Inoculation of caseum surrogate	2 hr
	7	Preadaptation phase	6 weeks
	8‐12	Preparation of MBC assay	3 hr
	13	Period of drug exposure	7 days
	14‐15	CFU plating and colony growth	4 weeks
	16‐19	CFU enumeration and calculation	2‐3 hr

### Author Contributions

**Min Xie**: Data curation; formal analysis; investigation; validation; visualization. **Paulina Osiecki**: Formal analysis; investigation; validation. **Suyapa Rodriguez**: Formal analysis; investigation. **Véronique Dartois**: Conceptualization; data curation; methodology; resources; supervision. **Jansy Sarathy**: Conceptualization; data curation; formal analysis; investigation; methodology; project administration; resources; supervision; validation; visualization; writing—original draft; writing—review and editing.

### Conflict of Interest

The authors declare no conflict of interest.

## Data Availability

The data, tools, and material (or their source) that support the protocols are available from the corresponding author upon reasonable request.
